# Missed Radiological Diagnosis of Otosclerosis in High-Resolution Computed Tomography of the Temporal Bone—Retrospective Analysis of Imaging, Radiological Reports, and Request Forms

**DOI:** 10.3390/jcm12020630

**Published:** 2023-01-12

**Authors:** Mohamed Bassiouni, Hans-Christian Bauknecht, Gloria Muench, Heidi Olze, Julian Pohlan

**Affiliations:** 1 Department of Otorhinolaryngology, Charité—Universitätsmedizin Berlin, Corporate Member of Freie Universität Berlin, Humboldt-Universität zu Berlin, and Berlin Institute of Health, 10117 Berlin, Germany; 2 Institute of Neuroradiology, Charité—Universitätsmedizin Berlin, Corporate Member of Freie Universität Berlin, Humboldt-Universität zu Berlin, and Berlin Institute of Health, 10117 Berlin, Germany; 3 Department of Diagnostic Radiology, Charité—Universitätsmedizin Berlin, Corporate Member of Freie Universität Berlin, Humboldt-Universität zu Berlin, and Berlin Institute of Health, 10117 Berlin, Germany; 4 Berlin Institute of Health, Charité—Universitätsmedizin Berlin, 10117 Berlin, Germany

**Keywords:** otosclerosis, diagnostic imaging, computed tomography, hearing loss

## Abstract

Objectives: Several studies reported low detection rates of otosclerosis in high-resolution computed tomography (HRCT), especially when the scans were reviewed by non-specialized general radiologists. In the present study, we conducted a retrospective review of the detection of otosclerosis in HRCT by general radiologists and the impact of inadequately filled radiological request forms on the detection rate. Methods: Retrospective analysis of hospital records, HRCT reports, and radiological referral notes of 40 patients who underwent stapedotomy surgery for otosclerosis. HRCT imaging data sets were retrospectively reviewed by a blinded experienced neuroradiologist, whose reading served as the gold standard. Results: General radiologists reading HRCT scans had an overall detection rate of otosclerosis of 36.1% in this cohort (13 of 36 available HRCT reports). The neuroradiologist had a much higher detection rate of 82.5% (33 of 40 cases). Interobserver agreement between the general radiologists and the subspecialist neuroradiologist was poor (Cohen’s kappa κ = 0.26). General radiologists missed the diagnosis in 15 of the 33 CT-positive scans, corresponding to a missed diagnosis rate of 45.4%. There was a highly significant association between a missed diagnosis and the lack of an explicitly mentioned clinical suspicion of otosclerosis in the request forms (Pearson’s chi-squared test, *p* < 0.005). Conclusion: The diagnosis of otosclerosis is frequently missed by radiologists on HRCT scans of the temporal bone in a clinical setting. Possible reasons include a relative lack of experience of general radiologists with temporal bone imaging as well as the failure of clinicians to unambiguously communicate their suspicion of otosclerosis.

## 1. Introduction

Otosclerosis is a common middle ear pathology, resulting in progressive conductive or mixed hearing loss [1]. The condition is characterized by the occurrence of spongiotic and sclerotic lesions of the otic capsule bone, which mechanically interfere with the stapes’ mobility [2]. The diagnosis is typically based on clinical suspicion and audiometric findings [3]. The standard surgical treatment is currently considered to be the stapedotomy procedure, which involves the fenestration of the stapes footplate and insertion of a prosthesis that conducts sound from the incus into the inner ear [4]. The hearing results reported in the literature are generally favorable, with good success rates regardless of the fenestration technique and prosthesis variables [5,6,7,8].

Traditionally, imaging used to play a minor role in establishing the diagnosis, but is now increasingly employed to evaluate the extent of otosclerosis, predict possible complications, and to facilitate the planning of surgical treatment [9,10,11,12]. In addition, imaging studies can rule out other differential diagnoses before surgery [13]. Non-contrast high-resolution computed tomography (HRCT) has been established as the gold standard to visualize subtle histopathological alterations typical for otosclerosis [9,10,14,15,16]. The most common presentation is fenestral otoslcerosis, which is characterized by a small area of radiolucency at the fistula ante fenestram anterior to the oval window. The radiolucency may be difficult to detect, and multiple studies report a low detection rate of otosclerosis in CT scans, resulting in low sensitivity but high specificity [16]. However, the published sensitivity rates vary widely, partly due to differences in image quality, slice thickness, and study protocols [16]. Additionally, general radiologists were shown to have lower detection rates of otosclerosis compared to experienced neuroradiologists or specialized head and neck radiologists [12,17], suggesting that general radiologists are more likely to miss the diagnosis on CT images.

This study aimed at analyzing the rate of missed diagnoses of otosclerosis in preoperative CT scans obtained in a population of 40 patients who subsequently underwent primary stapedotomy for otosclerotic stapes fixation. The factors influencing the likelihood of missed diagnosis were evaluated.

## 2. Materials and Methods

The local ethics committee approved this retrospective study (approval number EA4/090/20) without requiring informed consent in accordance with the national and institutional regulations. The study involved a retrospective analysis of the radiology reports of 40 high-resolution temporal bone computed tomography scans (HRCT) obtained in 40 patients who underwent primary stapedotomy surgery for otosclerosis in our Department of Otorhinolaryngology between January 2017 and December 2021 with intraoperative confirmation of otosclerotic stapes fixation and the exclusion of other differential diagnoses. The study only included patients who underwent preoperative HRCT examination. Exclusion criteria were the absence of a preoperative HRCT scan, revision surgery, and presence of other middle ear pathologies. The HRCT request forms, completed by the referring clinicians, were retrieved and reviewed with respect to the provided clinical data, particularly data pertaining to the suspected diagnosis of otosclerosis.

In this study population, 12 patients had already undergone a CT scan in other community-based radiology practices before they first presented to our center. The external CT scans were performed with a variety of protocols and slice thicknesses, ranging from 0.3 to 1.3 mm. The remaining 28 patients underwent HRCT in our university hospital’s Department of Radiology using either a Toshiba Aquilion ONE (Toshiba Medical Systems, Nasu, Japan) or a General Electric revolution scanner (General Electric Healthcare, Wauwatosa, WI, USA) with a slice thickness of 0.6 mm. Eight of the twelve external radiology reports and request forms could be obtained for review. All 28 internal radiology reports and request forms could be retrieved and reviewed. As the gold standard, the CT images were reviewed by an experienced neuroradiologist, who was blinded to the results of the initial clinical readings by the general radiologists.

Interobserver agreement was assessed by comparing the original clinical reports with the findings identified in a blinded retrospective reading by an experienced neuroradiologist. The request forms and radiology reports were retrospectively analyzed to identify possible factors contributing to the missed radiological diagnosis.

All data were collected in Excel tables for descriptive statistics. Further statistical analysis was performed using JMP (Version 15.1; SAS Institute, Cary, NC, USA). Pearson’s chi-squared test was used to analyze correlations. A *p*-value of <0.05 was considered statistically significant.

## 3. Results

### 3.1. Detection of Otosclerosis in Temporal Bone Computed Tomography

**Figure 1:**
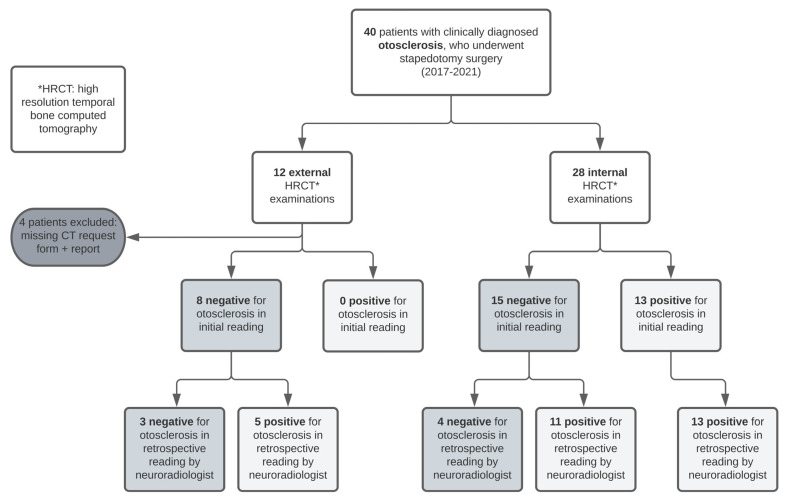
Flowchart summarizing the results of the initial readings by internal and external general radiologists as well as the second readings of the neuroradiologist.

Thirteen of the forty computed tomography (CT) scans included in our retrospective analysis were interpreted as positive for otosclerosis by the general radiologists. Twenty-three reports did not report the finding of otosclerosis, and four external CT reports could not be obtained. The image sets from all 40 CT scans were obtained and reviewed by a subspecialist neuroradiologist. Overall, general radiologists had a detection rate of 36.1% (13 out of 36 available reports). Internal general radiologists had a detection rate of 46.4% (13 of 28 internal CT reports). None of the available eight external CT reports reported a finding of otosclerosis. Of those eight negative external reports, three remained negative after the second reading by the neuroradiologist (Figure 1). Two of those three “true negative” CT scans had a slice thickness of 0.6 mm, while one had a slice thickness of 1.3 mm, which can be considered insufficient for the detection of otosclerosis.

The neuroradiologist had a detection rate of 82.5% (33 positive cases of 40). An illustrative summary of the results of the initial readings of general radiologists and the second readings of the subspecialist neuroradiologist is included in Figure 1. Analysis of the operative notes of the true “CT-negative” cases revealed a typical otosclerotic stapes fixation, with similar intraoperative findings among the CT-negative and CT-positive groups. This finding suggests that the radiological detectability of otosclerosis did not correlate with the clinical or surgical findings in our cohort.

**Figure 2:**
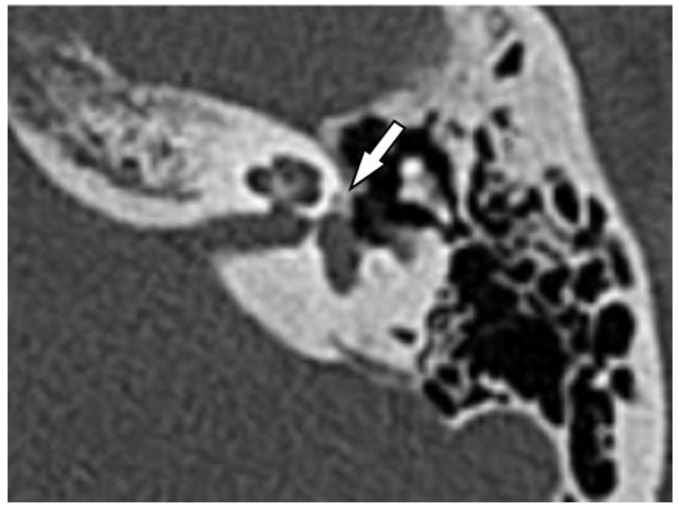
Non-contrast high-resolution temporal bone computed tomography (HRCT) image in the axial plane showing a temporal bone with surgically confirmed otosclerosis. The white arrow indicates a fenestral otosclerotic focus that was detected in the HRCT images by the subspecialist neuroradiologist in the second reading but missed by the general radiologist in the clinical routine reading.

Of the 33 scans that were interpreted as positive for otosclerosis by the neuroradiologist, 15 were rated negative by general radiologists, corresponding to a miss rate of 45.4% by general radiologists (Figure 1). An exemplary CT image of a fenestral otosclerotic lesion that was missed in the initial reading by the general radiologist is shown in Figure 2. Overall, there was poor interobserver agreement between the neuroradiologist and the general radiologists (Cohen’s kappa κ = 0.26). Thus, the detection rate of otosclerosis in HRCT appears to be lower for general radiologists compared to subspecialized neuroradiologists, resulting in a substantial rate of missed diagnoses in clinical practice.

### 3.2. Review of CT-Request Forms

Analysis of the relation between the information available to the general radiologists and the likelihood of a missed diagnosis of otosclerosis revealed that, in 10 of the total 36 available request forms, the ordering clinician did not explicitly mention otosclerosis as suspected diagnosis, but rather requested a CT scan for routine preoperative planning and anatomical mapping or for evaluation of hearing loss. Seven of the fifteen cases, in which the general radiologists did not report the radiological diagnosis of otosclerosis, were associated with a request form that did not explicitly mention otosclerosis as suspected clinical diagnosis. Statistical analysis revealed a highly significant association between a missed radiological diagnosis and the lack of explicit mention of otosclerosis in the request forms (Pearson’s chi-squared test < 0.005). These findings indicate that the failure to clearly communicate the suspected diagnosis in request forms may contribute to a low detection rate of otosclerosis in CT scans.

## 4. Discussion

The general rate of radiological errors reported in the literature is highly variable and may reach up to 30% or higher [18]. About 70% percent of such errors are accounted for by radiologists missing the pathology [18]. The subtle fenestral otosclerotic lesions investigated here may be particularly susceptible to such radiological misses, [14] especially if not explicitly mentioned in the request form by the ordering clinicians. One systematic review of level III evidence studies reported an average sensitivity rate of high-resolution computed tomography (HRCT) scans for otosclerosis of only 58% [16]. In a previous study, we detected a lack of correlation between the clinical phenotype and the radiological detectability of otosclerosis in CT scans [19]. Analysis of our operative notes revealed no remarkable difference in the intraoperative findings of the CT-negative cases, which contrasts with some other previous studies that reported a higher likelihood of intraoperative complications in CT-negative or doubtful cases [20]. In the present study, we aimed at identifying possible non-patient-related factors that might contribute to the low detection rate of otosclerosis in routine CT scans.

Earlier studies already showed that the detection rate of otosclerosis in CT scans is lower for general radiologists than for dedicated subspecialists [12,17]. Consistent with these reports, the present study showed poor interobserver agreement between general radiologists and the experienced neuroradiologist. General radiologists missed 45% of otosclerosis cases that were diagnosed by the neuroradiologist. In a recent study, Maxwell and colleagues [12] reported a similar discrepancy, with a subspecialist neuroradiologist in a tertiary referral center achieving a detection rate almost double that of the local general radiologists in the community. One solution to improve detection rates in future would be to mandate that all CT scans for the evaluation of otosclerosis be read or supervised by subspecialists. Another option that has been proposed is to include cone-beam CT with ultra-thin slice reconstruction [21,22,23,24]. However, it is important to note that radiological misses cannot be completely eliminated by improving scanning protocols or image quality, since the responsible radiologist may still overlook the lesion. Therefore, the use of deep learning algorithms may help in detecting subtle otosclerotic foci in routine HRCT scans [25]. Another solution would be to include the antefenestral region in all structured HRCT reports and check lists [26,27], which will drive readers to address the presence versus absence of otosclerosis in all scans.

Perhaps the most direct approach to mitigate the low detection rate of otosclerosis by general radiologists would be to communicate the clinical data accurately and thoroughly in CT request forms. Radiological request forms are an integral part of clinician–radiologist communication [28,29,30]. Inadequately completed request forms are a well-known concern, and are associated with diagnostic errors as well as unhelpful or unnecessary scans [28,29,30]. In the present study, insufficient request forms were significantly correlated with a higher likelihood of missed diagnoses. Awareness should be raised among clinicians about the importance of precise and detailed requests.

The main limitations of this study include the retrospective design, limited sample size and the variation in the scanning protocols and slice thicknesses of the external CT scans. Future studies should include a prospective design and larger patient cohorts to better study the factors involved in radiological missed diagnoses, as a step towards improving the diagnostic accuracy of CT imaging in otosclerosis. In the patient population investigated here, the missed diagnoses of otosclerosis had no harmful consequences for patients, since the pathology was diagnosed and treated intraoperatively. Nevertheless, efforts should be made to improve the preoperative diagnostic workup and to thus ensure better interdisciplinary treatment of patients with hearing loss. A misdiagnosis, or delay in diagnosis, may lead to inadequate patient counseling, unnecessary diagnostic tests, and eventually to treatment delay. Training should be offered to general radiologists, who infrequently interpret HRCT of the temporal bone.

## 5. Conclusions

The diagnosis of otosclerosis is frequently missed by radiologists on high-resolution computed tomography (HRCT) scans of the temporal bone. Possible reasons include the relative lack of experience of general radiologists with temporal bone neuroimaging as well as the failure of clinicians to unambiguously communicate the suspected diagnosis. To mitigate this problem, proposed solutions may include continuing the education of general radiologists by subspecialists, optimizing image and scan parameters, and inclusion of otosclerosis in structured check lists of HRCT reporting. Referring clinicians should provide detailed high-quality clinical information in HRCT request forms, including a suspected diagnosis of otosclerosis.

## Data Availability

More detailed data are available upon personal request. Due to privacy restrictions not all raw data are made publicly available.
